# Dual-allele heterozygous mutation of DNAH5 gene in a boy with primary ciliary dyskinesia: A case report

**DOI:** 10.1097/MD.0000000000036271

**Published:** 2023-12-29

**Authors:** Yu Shi, Qihong Lei, Qing Han

**Affiliations:** a Department of Respiratory, Children’s Hospital of Nanjing Medical University, Nanjing, China.

**Keywords:** DNAH5, gene mutation, next-generation sequencing, primary ciliary dyskinesia, transmission electron microscope

## Abstract

**Rationale:**

To analyze clinical and imaging features, ciliary structure and family gene mutation loci of a primary ciliary dyskinesia (PCD) boy with a dual-allele heterozygous mutation of DNAH5.

**Patient concerns:**

Clinical data of the proband and relatives. Electronic bronchoscopy, transmission electron microscope (TEM) of the cilia and next-generation sequencing (NGS) were performed. PCD-related DNAH5 exon mutation sites were searched.

**Diagnoses:**

A 10-year and 10-month-old boy was hospitalized due to “recurrent cough, expectoration, sputum and shortness of breathing after activity for over 7 years, and aggravated for 1 week.” Moderate and fine wet rales were detected in bilateral lungs. Clubbing fingers and toes were observed. In local hospitals, he was diagnosed with *Mycoplasma pneumoniae* infection and *Streptococcus pneumoniae* was cultured.

**Interventions:**

Pulmonary function testing showed mixed ventilation dysfunction and positive for bronchial dilation test. Imaging examination and fiberoptic bronchoscopy revealed transposition of all viscera, bilateral pneumonia, and bronchiectasis. TEM detected no loss of the outer dynein arms. NGS identified 2 mutations (c.4360C>T, c.9346C>T) in the DNAH5 gene inherited from healthy parents.

**Outcomes:**

According to literature review until 2022, among 144 exon gene mutations causing amino acid changes, C>T mutation is the most common in 44 cases, followed by deletion mutations in 30 cases. Among the amino acid changes induced by gene mutation, terminated mutations were identified in 89 cases.

**Lessons:**

For suspected PCD patients, TEM and NGS should be performed. Prompt diagnosis and treatment may delay the incidence of bronchiectasis and improve clinical prognosis.

## 1. Introduction

Primary ciliary dyskinesia (PCD) is an autosomal recessive monogenic disease caused by gene abnormality. Approximately 50% of PCD patients develop abnormal changes in organ laterality during the first-trimester pregnancy. The incidence of male and female is basically equivalent.^[1]^ PCD has an estimated prevalence rate of 1:15–300,000 live births.^[2]^

More than 200 genes have been confirmed to encode ciliary proteins,^[3]^ and nearly 40 PCD-associated pathogenic genes have been reported according to Online Mendelian Inheritance in Man (OMIM).^[4]^ Among them, DNAH5 (OMIM:603335) gene mutation is the most common, accounting for 15% to 21%.^[5]^ There is correlation and heterogeneity between the genotype and clinical phenotype of PCD.^[6]^ No gold standard has been reached for the diagnosis of the heterogeneity of clinical phenotype, leading to delays in the diagnosis and treatment of PCD, especially for those with mild symptoms, but without abnormal visceral laterality.^[7]^

Only 2 PCD cases of DNAH5 gene mutation have been reported in China until June, 2018.^[8,9]^ Here, we reported 1 case of Kartagener syndrome, a variant of PCD, associated with a dual-allele heterozygous mutation of DNAH5. Clinical features, ciliary structure and loci of family gene mutations were described as follows.

## 2. Case presentation

This study was approved by the Ethics Committee of Children’s Hospital of Nanjing Medical University. Written informed consents were obtained from the guardians in this study.

The proband was a boy, aged 10 years and 10 months, developed recurrent respiratory infections and visceral transposition after age 3. His parents and younger brother were healthy. He was admitted to hospital (January 31, 2018) because of “repeated cough, expectoration, shortness of breath after exercise for 7 years, and aggravated for 1 week.” CT scan revealed the inflammation of bilateral maxillary sinus and ethmoid sinus, inferior nasal congestion, lung inflammation, atelectasis of the middle lobe and lingular segment and bronchiectasis and total visceral transposition.

Purulent rhinorrhea and poor ventilation were found. Mild tenderness was palpable in bilateral maxillary sinuses. Pharyngeal congestion, bilateral tonsillitis II and slight shortness of breath were found. Moderate to slight moist rales were audible in both inferior lungs. Slight clubbed fingers and toes were noted. C-reactive protein was detected as 11 mg/L, white blood cell count was 16.84 × 10^9^/L, neutrophil percentage was 69.8%, Hb was 131g/L, Plt was 343 × 10^9^/L. PO_2_ was 85 mm Hg and PCO_2_ of 37.8 mm Hg. He tested positive for blood MP-IgM, and sputum MP-DNA was ranged from 2.33 × 10^3^ to 8.17 × 10^5^ copies. *Streptococcus pneumoniae* was detected in 1 sputum culture test (+++). He was positive for bronchodilation test. The lowest values of PEF and FEVl were 56.7% and 64.0%. The variation rate of PEF was 15.5%. The FEV1/FVC ratio was 56.7%, respectively. Mild mixed ventilation dysfunction was confirmed. FVC/estimated FVC ratio was 56.7% and the variation rate of FVC was 19.2%.

**Figure 1.**
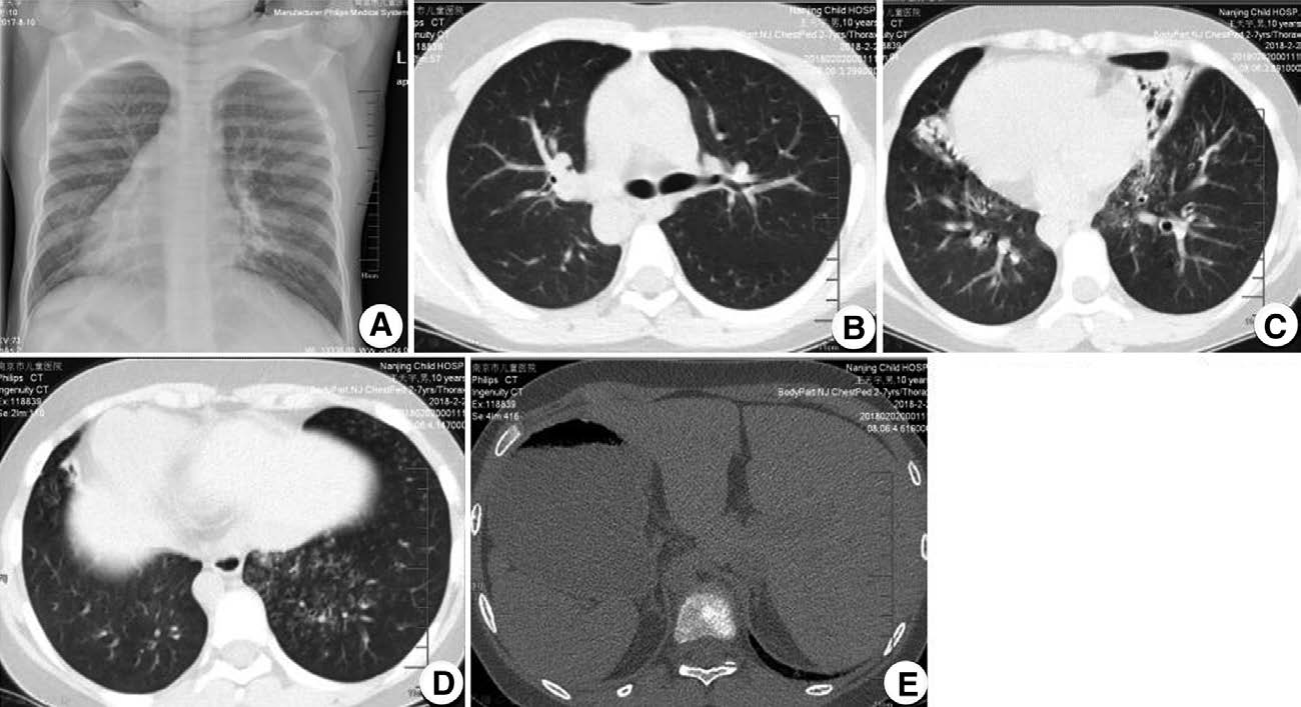
B-mode ultrasound and CT scan showing transposition of the heart, bronchus, stomach, liver, and spleen (A, B, and E); The volume of the right ligule and the left middle lung was shrank, and bronchiectasis was seen. Multiple small patches were noted in the lower lobes.

**Figure 2.**
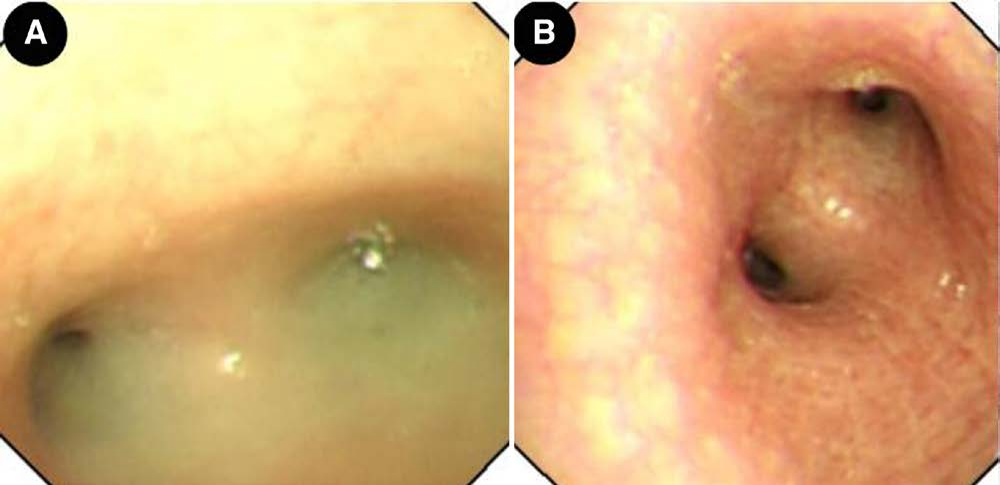
Fiberoptic bronchoscopy showing a large number of sticky secretions at the opening of the main bronchus (A) and fish bone-like changes and small ulcers in the middle of the middle left bronchus (B).

**Figure 3.**
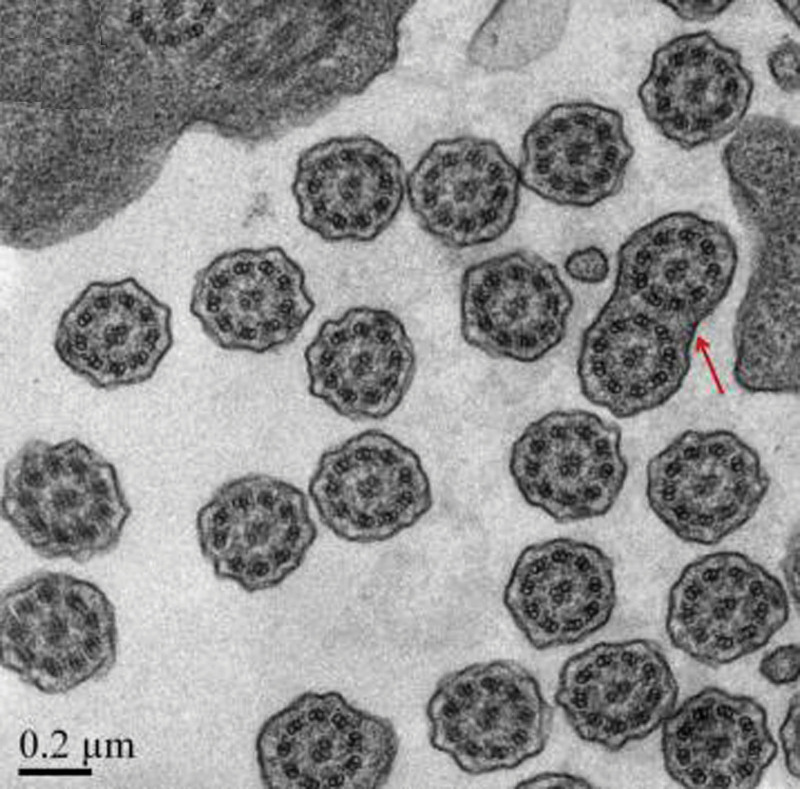
Transmission electron microscopy revealing no abnormal structure of 9 + 2 and no ODA in the transverse section. Amalgamation of cilium (red arrow). ODA = outer dynein arm.

**Figure 4.**
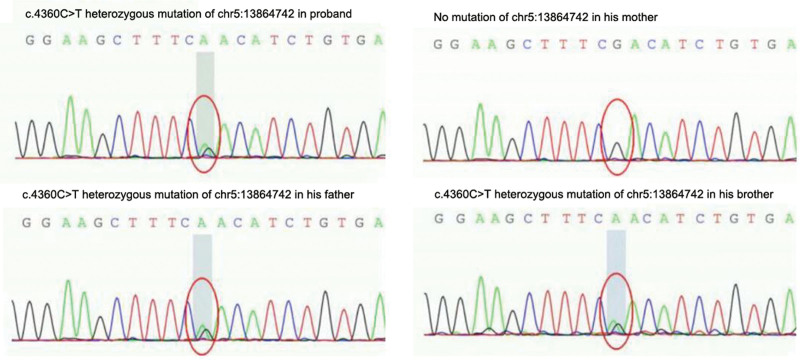
Targeted next-generation sequencing indicating the mutation site 1: DNAH5, chr5:13864742, c.4360C>T inherited from his father.

**Figure 5.**
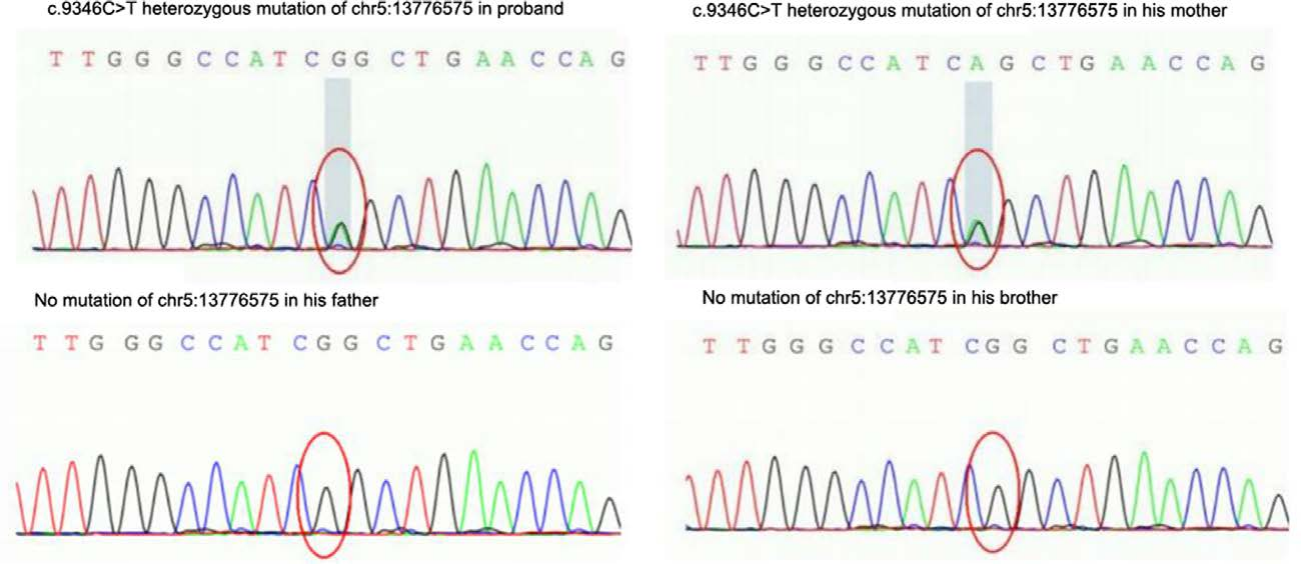
Targeted next-generation sequencing indicating the mutation site 2: DNAH5, chr5:13776575, c.9346C>T inherited from his mother.

Total visceral transposition was found. Bilateral lung pneumonia was observed, as illustrated in Figure 1. Fiberoptic bronchoscopy displayed sticky secretions at the opening of the main bronchus and fish bone-like changes and small ulcers in the middle of the middle left bronchus (Fig. 2). Transmission electron microscope revealed no abnormal structural changes of 9 + 2 and no outer dynein arm in the transverse section. Amalgamation of cilium was denoted by the red arrow in Figure 3. Targeted next-generation sequencing (NGS) verified the mutation site 1:DNAH5, chr5:13864742, c.4360C>T, leading to amino acid changes of p.R1454X, inherited from his father (Fig. 4). The mutation site 2:DNAH5, chr5:13776575, c.9346C>T was inherited from his mother (Fig. 5).

He was eventually diagnosed with PCD, Kartagener syndrome and severe pneumonia. After 10-d intravenous injection of loxceftazidime sodium and erythromycin, sequential anti-infection therapy with oral azithromycin, mucosolvan, propafenone, back slapping, and postural drainage, the proband was discharged after 14-d hospitalization. During 6-month follow-up, the proband’s condition remained stable.

Relevant studies were searched using the keywords of “PCD,” “gene,” and “DNAH5” from CNKI, Wanfang Database, PubMed, HGMD, and OMIM from the inception of databases to November, 2022. Nineteen articles with relatively complete references were obtained. One hundred forty-four mutation sites of DNAH5 exon (cDNA) were associated with PCD (Table 1), and PCD caused by single mutation of DNAH5 exon was not reported.

**Table 1:** Mutation sites (c.) and amino acid changes (p.) in PCD-associated DNAG5 exon.

No.	Exon	Mutation site	Amino acid changes	No.	Exon	Mutation site	Amino acid changes	No.	Exon	Mutation site	Amino acid changes
1	2	c.232C>T	p.R78X	49	33	c.5588delT	p.F1863SfsX8	97	52	c.8887C > G	p.Q2949E
2	2	c.252T>G	p.Y84X	50	33	c.5599_5600insC	p.I1867PfsX35	98	52	c.8910_8911delATinsG	p.S2970LfsX7
3	5	c.670C>T	p.R224X	51	33	c.5647C > T	R1883X	99	53	c.8998C > T	p.R3000X
4	5	c.717_729delCTACTTGACTCTA	p.D239EfsX11	52	35	c.5983C > T	p.R1995X	100	53	c.8999G > A	p.A3000Q
5	6	c.832delG	p.A278RfsX27	53	35	c.6037C > T	p.R2013X	101	53	c.9018C > T	splicing-mut.
6	6	c.894C>G	p.N298K	54	36	c.6086delG	p.G2029VfsX25	102	53	c.9040C > T	p.R3000X
7	8	c.1108A>T	p.I370F	55	37	c.6132delT	p.A2045Ufs	103	53	c.9101delG	p.G3034VfsX23
8	9	c.1206T>A	p.N402K	56	36	c.6249G > A	p.G2021EfsX12	104	55	c.9213delC	p.H3071QfsX5
9	9	c.1232A>G	p.Y411C	57	37	c.6304C > T	p.R2102C	105	54	c.9286C > T	p.R3096X
10	10	C.1427_1428delTT	p.F476SfsX26	58	37	c.6335_6336insT	p.E2112HfsX10	106	55	c.9346C > T	p.R3116X
11	10	c.1432C>T	p.R478X	59	37	c.6343delA	p.I2115X	107	55	c.9365delT	p.L3122X
12	11	c.1489C>T	p.Q497X	60	37	c.6647delA	p.K2216RfsX20	108	55	c.9427A > T	p.K3143X
13	11	c.1619T>C	p.F540L	61	41	c.6763C > T	p.A2255X	109	55	c.9799C > T	p.E3267X
14	11	c.1627C>T	p.Q543X	62	41	c.6786delG	p.S2264VfsX2	110	57	c.10048T > C	p.S3350P
15	12	c.1645A>G	p.N549D	63	40	c.6791G > A	p.S2264N	111	59	c.10226G > C	p.W3409S
16	12	c.1667A>G	p.D556G	64	41	c.6932_6935delACTG	p.D2311GfsX14	112	61	c.10363G > T	p.Q3455X
17	12	c.1730G>C	p.N549_R577delfsX5	65	42	c.7039G > A	p.E2347K	113	60	c.10365G > C	p.Q3455H
18	13	c.1828C>T	p.Q610X	66	43	c.7387C > T	p.E2463X	114	60	c.10384C > T	p.E3462X
19	15	c.2291C>A	p.S764X	67	44	c.7429C > T	p.Q2463X	115	61	c.10426C > T	p.Q3462X
20	16	c.2578 + 1 + T.C	p.A2639TfsX19	68	44	c.7502G > C	p.R2501P	116	61	c.10441C > T	p.R358X
21	17	c.2686_2689dup	p.E897GfsX4	69	44	c.7550_7556delAGCTGCC	p.E2517GfsX52	117	61	c.10555G > C	p.G3519R
22	18	c.2772delC	p.L925X	70	44	c.7561_7573delCCAGCGGGGCCCG	p.2521GfsX46	118	62	c.10615C > T	p.R3539C
23	19	c.3036_3041delAGCG	p.V1014LfsX20	71	45	c.7624T > C	p.W2542A	119	62	c.10616G > A	p.R3539H
24	23	c.3484C>T	p.Q1162X	72	45	c.7778C > T	p.G2593E	120	62	c.10813G > A	p.D3605N
25	24	c.3712G>T	p.E1238X	73	47	c.7888A > T	p.R2630W	121	62	c.10815delT	p.P3606HfsX23
26	25	c.3905delT	p.L1302RfsX19	74	47	c.7897_7902delAGAG	p.E2633AfsX18	122	65	c.11308A > G	p.S3770G
27	26	c.4348C>T	p.Q1450X	75	47	c.7914_7915insA	p.R2639TfsX19	123	65	c.11428_11434delACTCA	p.N3810SfsX21
28	26	c.4355 + 1G>A	p.I1855NfsX5	76	47	c.7915C > T	p.R2639X	124	66	c.11528C > T	p.S3843L
29	27	c.4360C>T	p.R1454X	77	48	c.8012A > G	p.Q2701A	125	68	c.11583C > A	p.S3861R
30	27	c.4361G>A	p.R1454Q	78	48	c.8029C > T	p.R2677X	126	69	c.12009G > A	p.W4003X
31	28	c.4660G>T	p.E1554X	79	48	c.8030G > A	p.R2677Q	127	70	c.12107G > A	p.W4036X
32	29	c.4830dup	p.W1611MfsX47	80	48	c.8092_8097delGTGGAC	p.V2698_D2699del	128	70	c.12265C > T	p.E4089X
33	29	c.4837C>T	p.E1613X	81	48	c.8141delA	p.N2714MfsX30	129	71	c.12397G > T	p.E4133X
34	29	c.4879C>T	p.Q1613X	82	48	c.8147T > C	p.I2716T	130	72	c.12614G > T	p.G4205V
35	31	c.5034C>A	p.C1678X	83	48	c.8167C > T	p.Q2723X	131	72	c.12617G > A	p.W4206X
36	31	c.5130A>C	p.K1710N	84	48–50	Loss of heterozygosity	Not available	132	72	c.12705G > T	p.K4235N
37	31	c.5130_5131insA	p.R1711TfsX36	85	49	c.8314C > T	p.R2772X	133	74	c.12813G > A	p.W4271X
38	31	c.5146C>T	p.R1716W	86	49	c.8383C > T	p.R2795X	134	75	c.13194_13197delCAGA	p.D4398EfsX16
39	31	c.5147G>T	p.R1716L	87	49	c.8396G > C	p.R2799P	135	76	c.13426C > T	p.R4476X
40	31	c.5172A>C	p.K1710N	88	49	c.8404C > T	p.Q2802X	136	76	c.13458dupT	p.?
41	31	c.5177T>C	p.L1726P	89	49	c.8440_8447delGAACCAAA	p.2814fsX1	137	76	c.13458_13459insT	p.N4487fsX1
42	32	c.5281C>T	p.R1761X	90	51	c.8498G > A	p.A2833H	138	77	c.13486C > T	p.R4496X
43	32	c.5367delT	p.N1790IfsX14	91	50	c.8485G > T	p.V2829F	139	78	c.13595G > T	p.G4532V
44	32	c.5482C>T	p.Q1828X	92	50	c.8497C > T	p.R2833C	140	78	c.13633T > C	p.W4545R
45	33	c.5545G>A	p.A1849T	93	50	c.8497C > G	p.R2833G	141	79	c.13729G > A	p.R4577X
46	33	c.5557A>T	p.K1853X	94	50	c.8498G > A	p.R2833H	142	79	c.13760A > G	p.Y4587C
47	33	c.5563dupA	p.I1855NfsX6	95	50	c.8528T > C	p.F2843S	143	79	c.13778C > T	p.T4593M
48	33	c.5563_5564insA	p.I1855X	96	50	c.8642C > G	p.A2881G	144	79	c.13837delG	p.V4613X

## 3. Discussion

For the proband in this study, NGS detected dual compound heterozygous mutation sites in DNAH5 exon regions: c.4360C>T and c.9346C>T, among which c.4360C>T has been reported as pathogenic in HGMDpro database,^[8]^ and c.9346C>T has been classified as pathogenic by the American College of Medical Genetics and Genomics.^[10]^ Both of them are stop mutations, suggesting that these 2 gene mutations directly cause clinical manifestations of the proband. Although nasal nitric oxide, high-speed digital video imaging and immunofluorescence microscopy were not carried out due to limited laboratory conditions, symptoms, signs, lung function, high-resolution CT scan, TEM, and NGS all supported the diagnosis of Katargener syndrome in this proband.

These 2 heterozygous mutations of the proband were inherited from his parents. However, his parents and younger brother with single-gene heterozygous mutation presented with no relevant clinical manifestations, suggesting that PCD induced by dual-allele compound heterozygous mutation in DNAH5 exon probably follows the autosomal recessive inheritance law,^[11]^ which is determined by 1 pathogenic gene and functions jointly by several modified genes.^[12]^ Zhang et al^[13]^ reported a 48-year-old woman with a single mutation of DNAH5 [c.9286C>T(p.R3096X)] complicated with a dual-allele heterozygous missense mutation of DNAH11, which led to Kartagener syndrome and overlapped non-ciliated moyamoya syndrome. In our study, although the parents and younger brother with single gene heterozygous mutation developed no relevant clinical manifestations, the ciliary function may have been affected to certain extent, which can still maintain relatively normal organ function.

The proband developed mixed ventilation dysfunction and positive bronchodilation test. No abnormal changes, such as hearing loss, retinitis pigmentosa, polycystic kidney, hydrocephalus, abnormal development of spleen, growth disorder and gastroesophageal reflux were found in the proband of our study. Approximately 30% of PCD patients have normal cilia on TEM, which can partly explain why no evident 9 + 2 structural abnormality was observed under TEM in the proband.

In the present study, the proband was confirmed to have MP infection during each admission to our hospital, whereas *S pneumoniae* was cultured in only 1 testing, which was consistent with the possibility of mixed infection reported by Xu et al,^[14]^ whereas inconsistent with the findings of Noone et al^[15]^ that the most common pathogens causing lung infection in PCD patients were *Haemophilus influenzae, S pneumoniae, Staphylococcus aureus, Pseudomonas aeruginosa, Escherichia coli*, etc.

## 4. Study limitations

Due to the small sample size, the findings in this single-case report remain to be validated by subsequent clinical trials with larger sample size.

## 5. Conclusion

For suspected PCD patients, TEM and NGS should be performed for accurate diagnosis. Prompt prevention and treatment may delay the incidence of bronchiectasis and improve clinical prognosis.

## Author contributions

**Conceptualization:** Yu Shi, Qihong Lei, Qing Han.

**Data curation:** Yu Shi, Qihong Lei, Qing Han.

**Investigation:** Yu Shi, Qihong Lei, Qing Han.

**Methodology:** Yu Shi, Qihong Lei, Qing Han.

**Writing – original draft:** Yu Shi, Qihong Lei, Qing Han.

**Writing – review & editing:** Yu Shi, Qihong Lei, Qing Han.
